# Supplementary material for: Heterozygous diploid and interspecies SCRaMbLEing
Source: Nat Commun. 2018 May 22;9:1934. doi: 10.1038/s41467-018-04157-0 (PMC5964232; doi:10.1038/s41467-018-04157-0)
Supplement: Supplementary file 1 — Supplementary Information [file 41467_2018_4157_MOESM1_ESM.pdf]

Supplementary Tables and Figures for:

Heterozygous diploid and interspecies SCRaMbLEing

Shen et al.

Supplementary Table 1: List of heterozygous diploid strains. Bold strains were used in this study.

Strain Name	Description	Genotype
**yMS401**	**synX and S288c**	*MAT***a***/α ura3Δ0/ura3::KanMX ho::tR(ccu)J/ho::Hyg lys2::NatMX/LYS2 SYN10/WT10*
**yMS402**	synX and UWOPS87-2421	*MAT***a***/α ura3Δ0/ura3::KanMX ho::tR(ccu)J/ho::Hyg lys2::NatMX/LYS2 SYN10/WT10*
**yMS403**	synX and 378604X	*MAT***a***/α ura3Δ0/ura3::KanMX ho::tR(ccu)J/ho::Hyg lys2::NatMX/LYS2 SYN10/WT10*
**yMS404**	synX and 273614N	*MAT***a***/α ura3Δ0/ura3::KanMX ho::tR(ccu)J/ho::Hyg lys2::NatMX/LYS2 SYN10/WT10*
**yMS405**	synX and YIIc17_E5	*MAT***a***/α ura3Δ0/ura3::KanMX ho::tR(ccu)J/ho::Hyg lys2::NatMX/LYS2 SYN10/WT10*
**yMS406**	synX and Y55	*MAT***a***/α ura3Δ0/ura3::KanMX ho::tR(ccu)J/ho::Hyg lys2::NatMX/LYS2 SYN10/WT10*
**yMS407**	synX and UWOPS83-787.3	*MAT***a***/α ura3Δ0/ura3::KanMX ho::tR(ccu)J/ho::Hyg lys2::NatMX/LYS2 SYN10/WT10*
**yMS408**	synX and SK1	*MAT***a***/α ura3Δ0/ura3::KanMX ho::tR(ccu)J/ho::Hyg lys2::NatMX/LYS2 SYN10/WT10*
**yMS409**	synX and BC187	*MAT***a***/α ura3Δ0/ura3::KanMX ho::tR(ccu)J/ho::Hyg lys2::NatMX/LYS2 SYN10/WT10*
**yMS410**	synX and YJM978	*MAT***a***/α ura3Δ0/ura3::KanMX ho::tR(ccu)J/ho::Hyg lys2::NatMX/LYS2 SYN10/WT10*
**yMS411**	synX and YJM981	*MAT***a***/α ura3Δ0/ura3::KanMX ho::tR(ccu)J/ho::Hyg lys2::NatMX/LYS2 SYN10/WT10*
**yMS412**	synX and YJM975	*MAT***a***/α ura3Δ0/ura3::KanMX ho::tR(ccu)J/ho::Hyg lys2::NatMX/LYS2 SYN10/WT10*
**yMS413**	synX and DBVPG 1373	*MAT***a***/α ura3Δ0/ura3::KanMX ho::tR(ccu)J/ho::Hyg lys2::NatMX/LYS2 SYN10/WT10*
**yMS414**	synX and DBVPG 1106	*MAT***a***/α ura3Δ0/ura3::KanMX ho::tR(ccu)J/ho::Hyg lys2::NatMX/LYS2 SYN10/WT10*
**yMS415**	synX and DBVPG 6765	*MAT***a***/α ura3Δ0/ura3::KanMX ho::tR(ccu)J/ho::Hyg lys2::NatMX/LYS2 SYN10/WT10*
**yMS416**	synX and L_1374	*MAT***a***/α ura3Δ0/ura3::KanMX ho::tR(ccu)J/ho::Hyg lys2::NatMX/LYS2 SYN10/WT10*
**yMS417**	synX and L_1528	*MAT***a***/α ura3Δ0/ura3::KanMX ho::tR(ccu)J/ho::Hyg lys2::NatMX/LYS2 SYN10/WT10*
**yMS418**	synX and DBVPG 6044	*MAT***a***/α ura3Δ0/ura3::KanMX ho::tR(ccu)J/ho::Hyg lys2::NatMX/LYS2 SYN10/WT10*
**yMS419**	synX and NCYC 110	*MAT***a***/α ura3Δ0/ura3::KanMX ho::tR(ccu)J/ho::Hyg lys2::NatMX/LYS2 SYN10/WT10*
**yMS420**	synX and UWOPS03-461.4	*MAT***a***/α ura3Δ0/ura3::KanMX ho::tR(ccu)J/ho::Hyg lys2::NatMX/LYS2 SYN10/WT10*
**yMS421**	synX and UWOPS05-217.3	*MAT***a***/α ura3Δ0/ura3::KanMX ho::tR(ccu)J/ho::Hyg lys2::NatMX/LYS2 SYN10/WT10*
**yMS422**	synX and UWOPS05-227.2	*MAT***a***/α ura3Δ0/ura3::KanMX ho::tR(ccu)J/ho::Hyg lys2::NatMX/LYS2 SYN10/WT10*
**yMS423**	**synX and Y12**	*MAT***a***/α ura3Δ0/ura3::KanMX ho::tR(ccu)J/ho::Hyg lys2::NatMX/LYS2 SYN10/WT10*

**yMS424**	synX and YPS606	*MAT**a**/α ura3Δ0/ura3::KanMX ho::tR(ccu)J/ho::Hyg lys2::NatMX/LYS2 SYN10/WT10*
**yMS425**	synX and YPS128	*MAT**a**/α ura3Δ0/ura3::KanMX ho::tR(ccu)J/ho::Hyg lys2::NatMX/LYS2 SYN10/WT10*
**yMS426**	**synVsynX and S288c**	*MAT**a**/α ura3Δ0/ura3::KanMX ho::tR(ccu)J/ho::Hyg lys2::NatMX/LYS2 SYN5/WT5 SYN10/WT10*
**yMS427**	synVsynX and UWOPS87-2421	*MAT**a**/α ura3Δ0/ura3::KanMX ho::tR(ccu)J/ho::Hyg lys2::NatMX/LYS2 SYN5/WT5 SYN10/WT10*
**yMS428**	synVsynX and 378604X	*MAT**a**/α ura3Δ0/ura3::KanMX ho::tR(ccu)J/ho::Hyg lys2::NatMX/LYS2 SYN5/WT5 SYN10/WT10*
**yMS429**	synVsynX and 273614N	*MAT**a**/α ura3Δ0/ura3::KanMX ho::tR(ccu)J/ho::Hyg lys2::NatMX/LYS2 SYN5/WT5 SYN10/WT10*
**yMS430**	synVsynX and YIIc17_E5	*MAT**a**/α ura3Δ0/ura3::KanMX ho::tR(ccu)J/ho::Hyg lys2::NatMX/LYS2 SYN5/WT5 SYN10/WT10*
**yMS431**	synVsynX and Y55	*MAT**a**/α ura3Δ0/ura3::KanMX ho::tR(ccu)J/ho::Hyg lys2::NatMX/LYS2 SYN5/WT5 SYN10/WT10*
**yMS432**	synVsynX and UWOPS83-787.3	*MAT**a**/α ura3Δ0/ura3::KanMX ho::tR(ccu)J/ho::Hyg lys2::NatMX/LYS2 SYN5/WT5 SYN10/WT10*
**yMS433**	synVsynX and SK1	*MAT**a**/α ura3Δ0/ura3::KanMX ho::tR(ccu)J/ho::Hyg lys2::NatMX/LYS2 SYN5/WT5 SYN10/WT10*
**yMS434**	synVsynX and BC187	*MAT**a**/α ura3Δ0/ura3::KanMX ho::tR(ccu)J/ho::Hyg lys2::NatMX/LYS2 SYN5/WT5 SYN10/WT10*
**yMS435**	synVsynX and YJM978	*MAT**a**/α ura3Δ0/ura3::KanMX ho::tR(ccu)J/ho::Hyg lys2::NatMX/LYS2 SYN5/WT5 SYN10/WT10*
**yMS436**	synVsynX and YJM981	*MAT**a**/α ura3Δ0/ura3::KanMX ho::tR(ccu)J/ho::Hyg lys2::NatMX/LYS2 SYN5/WT5 SYN10/WT10*
**yMS437**	synVsynX and YJM975	*MAT**a**/α ura3Δ0/ura3::KanMX ho::tR(ccu)J/ho::Hyg lys2::NatMX/LYS2 SYN5/WT5 SYN10/WT10*
**yMS438**	synVsynX and DBVPG 1373	*MAT**a**/α ura3Δ0/ura3::KanMX ho::tR(ccu)J/ho::Hyg lys2::NatMX/LYS2 SYN5/WT5 SYN10/WT10*
**yMS439**	synVsynX and DBVPG 1106	*MAT**a**/α ura3Δ0/ura3::KanMX ho::tR(ccu)J/ho::Hyg lys2::NatMX/LYS2 SYN5/WT5 SYN10/WT10*
**yMS440**	synVsynX and DBVPG 6765	*MAT**a**/α ura3Δ0/ura3::KanMX ho::tR(ccu)J/ho::Hyg lys2::NatMX/LYS2 SYN5/WT5 SYN10/WT10*
**yMS441**	synVsynX and L_1374	*MAT**a**/α ura3Δ0/ura3::KanMX ho::tR(ccu)J/ho::Hyg lys2::NatMX/LYS2 SYN5/WT5 SYN10/WT10*
**yMS442**	synVsynX and L_1528	*MAT**a**/α ura3Δ0/ura3::KanMX ho::tR(ccu)J/ho::Hyg lys2::NatMX/LYS2 SYN5/WT5 SYN10/WT10*
**yMS443**	synVsynX and DBVPG 6044	*MAT**a**/α ura3Δ0/ura3::KanMX ho::tR(ccu)J/ho::Hyg lys2::NatMX/LYS2 SYN5/WT5 SYN10/WT10*
**yMS444**	synVsynX and NCYC 110	*MAT**a**/α ura3Δ0/ura3::KanMX ho::tR(ccu)J/ho::Hyg lys2::NatMX/LYS2 SYN5/WT5 SYN10/WT10*
**yMS445**	synVsynX and UWOPS03-461.4	*MAT**a**/α ura3Δ0/ura3::KanMX ho::tR(ccu)J/ho::Hyg lys2::NatMX/LYS2 SYN5/WT5 SYN10/WT10*
**yMS446**	synVsynX and UWOPS05-217.3	*MAT**a**/α ura3Δ0/ura3::KanMX ho::tR(ccu)J/ho::Hyg lys2::NatMX/LYS2 SYN5/WT5 SYN10/WT10*
**yMS447**	synVsynX and UWOPS05-227.2	*MAT**a**/α ura3Δ0/ura3::KanMX ho::tR(ccu)J/ho::Hyg lys2::NatMX/LYS2 SYN5/WT5 SYN10/WT10*
**yMS448**	**synVsynX and Y12**	*MAT**a**/α ura3Δ0/ura3::KanMX ho::tR(ccu)J/ho::Hyg lys2::NatMX/LYS2 SYN5/WT5 SYN10/WT10*
**yMS449**	synVsynX and YPS606	*MAT**a**/α ura3Δ0/ura3::KanMX ho::tR(ccu)J/ho::Hyg lys2::NatMX/LYS2 SYN5/WT5 SYN10/WT10*
**yMS450**	synVsynX and YPS128	*MAT**a**/α ura3Δ0/ura3::KanMX ho::tR(ccu)J/ho::Hyg lys2::NatMX/LYS2 SYN5/WT5 SYN10/WT10*

**yMS501**	synX and CBS432	*MATa/α ura3Δ0/ura3::KanMX ho::tR(ccu)J/ho::Hyg lys2::NatMX/LYS2 SYN10/WT10*
**yMS502**	synX and T21.4	*MATa/α ura3Δ0/ura3::KanMX ho::tR(ccu)J/ho::Hyg lys2::NatMX/LYS2 SYN10/WT10*
**yMS503**	synX and Y7	*MATa/α ura3Δ0/ura3::KanMX ho::tR(ccu)J/ho::Hyg lys2::NatMX/LYS2 SYN10/WT10*
**yMS504**	synX and Q32.3	*MATa/α ura3Δ0/ura3::KanMX ho::tR(ccu)J/ho::Hyg lys2::NatMX/LYS2 SYN10/WT10*
**yMS505**	synX and Q59.1	*MATa/α ura3Δ0/ura3::KanMX ho::tR(ccu)J/ho::Hyg lys2::NatMX/LYS2 SYN10/WT10*
**yMS506**	synX and Q95.3	*MATa/α ura3Δ0/ura3::KanMX ho::tR(ccu)J/ho::Hyg lys2::NatMX/LYS2 SYN10/WT10*
**yMS507**	synX and S36.7	*MATa/α ura3Δ0/ura3::KanMX ho::tR(ccu)J/ho::Hyg lys2::NatMX/LYS2 SYN10/WT10*
**yMS508**	synX and Z1.1	*MATa/α ura3Δ0/ura3::KanMX ho::tR(ccu)J/ho::Hyg lys2::NatMX/LYS2 SYN10/WT10*
**yMS509**	synX and Y6.5	*MATa/α ura3Δ0/ura3::KanMX ho::tR(ccu)J/ho::Hyg lys2::NatMX/LYS2 SYN10/WT10*
**yMS510**	synX and Q62.5	*MATa/α ura3Δ0/ura3::KanMX ho::tR(ccu)J/ho::Hyg lys2::NatMX/LYS2 SYN10/WT10*
**yMS511**	synX and Q89.8	*MATa/α ura3Δ0/ura3::KanMX ho::tR(ccu)J/ho::Hyg lys2::NatMX/LYS2 SYN10/WT10*
**yMS512**	synX and Y9.6	*MATa/α ura3Δ0/ura3::KanMX ho::tR(ccu)J/ho::Hyg lys2::NatMX/LYS2 SYN10/WT10*
**yMS513**	synX and Q74.4	*MATa/α ura3Δ0/ura3::KanMX ho::tR(ccu)J/ho::Hyg lys2::NatMX/LYS2 SYN10/WT10*
**yMS514**	synX and Q69.8	*MATa/α ura3Δ0/ura3::KanMX ho::tR(ccu)J/ho::Hyg lys2::NatMX/LYS2 SYN10/WT10*
**yMS515**	synX and W7	*MATa/α ura3Δ0/ura3::KanMX ho::tR(ccu)J/ho::Hyg lys2::NatMX/LYS2 SYN10/WT10*
**yMS516**	synX and Q31.4	*MATa/α ura3Δ0/ura3::KanMX ho::tR(ccu)J/ho::Hyg lys2::NatMX/LYS2 SYN10/WT10*
**yMS517**	synX and Y8.5	*MATa/α ura3Δ0/ura3::KanMX ho::tR(ccu)J/ho::Hyg lys2::NatMX/LYS2 SYN10/WT10*
**yMS518**	synX and Z1.1	*MATa/α ura3Δ0/ura3::KanMX ho::tR(ccu)J/ho::Hyg lys2::NatMX/LYS2 SYN10/WT10*
**yMS519**	synX and Y8.1	*MATa/α ura3Δ0/ura3::KanMX ho::tR(ccu)J/ho::Hyg lys2::NatMX/LYS2 SYN10/WT10*
**yMS520**	synX and N-17	*MATa/α ura3Δ0/ura3::KanMX ho::tR(ccu)J/ho::Hyg lys2::NatMX/LYS2 SYN10/WT10*
**yMS521**	**synX and CBS 5829**	*MATa/α ura3Δ0/ura3::KanMX ho::tR(ccu)J/ho::Hyg lys2::NatMX/LYS2 SYN10/WT10*
**yMS522**	synX and KPN3829	*MATa/α ura3Δ0/ura3::KanMX ho::tR(ccu)J/ho::Hyg lys2::NatMX/LYS2 SYN10/WT10*
**yMS523**	synX and YPS138	*MATa/α ura3Δ0/ura3::KanMX ho::tR(ccu)J/ho::Hyg lys2::NatMX/LYS2 SYN10/WT10*
**yMS524**	synX and DBVPG 6304	*MATa/α ura3Δ0/ura3::KanMX ho::tR(ccu)J/ho::Hyg lys2::NatMX/LYS2 SYN10/WT10*
**yMS525**	synX and A12	*MATa/α ura3Δ0/ura3::KanMX ho::tR(ccu)J/ho::Hyg lys2::NatMX/LYS2 SYN10/WT10*
**yMS526**	synX and N-44	*MATa/α ura3Δ0/ura3::KanMX ho::tR(ccu)J/ho::Hyg lys2::NatMX/LYS2 SYN10/WT10*
**yMS527**	synX and IFO 1804	*MATa/α ura3Δ0/ura3::KanMX ho::tR(ccu)J/ho::Hyg lys2::NatMX/LYS2 SYN10/WT10*

**yMS528**	synVsynX and CBS432	*MATa/α ura3Δ0/ura3::KanMX ho::tR(ccu)J/ho::Hyg lys2::NatMX/LYS2 SYN5/WT5 SYN10/WT10*
**yMS529**	synVsynX and T21.4	*MATa/α ura3Δ0/ura3::KanMX ho::tR(ccu)J/ho::Hyg lys2::NatMX/LYS2 SYN5/WT5 SYN10/WT10*
**yMS530**	synVsynX and Y7	*MATa/α ura3Δ0/ura3::KanMX ho::tR(ccu)J/ho::Hyg lys2::NatMX/LYS2 SYN5/WT5 SYN10/WT10*
**yMS531**	synVsynX and Q32.3	*MATa/α ura3Δ0/ura3::KanMX ho::tR(ccu)J/ho::Hyg lys2::NatMX/LYS2 SYN5/WT5 SYN10/WT10*
**yMS532**	synVsynX and Q59.1	*MATa/α ura3Δ0/ura3::KanMX ho::tR(ccu)J/ho::Hyg lys2::NatMX/LYS2 SYN5/WT5 SYN10/WT10*
**yMS533**	synVsynX and Q95.3	*MATa/α ura3Δ0/ura3::KanMX ho::tR(ccu)J/ho::Hyg lys2::NatMX/LYS2 SYN5/WT5 SYN10/WT10*
**yMS534**	synVsynX and S36.7	*MATa/α ura3Δ0/ura3::KanMX ho::tR(ccu)J/ho::Hyg lys2::NatMX/LYS2 SYN5/WT5 SYN10/WT10*
**yMS535**	synVsynX and Z1.1	*MATa/α ura3Δ0/ura3::KanMX ho::tR(ccu)J/ho::Hyg lys2::NatMX/LYS2 SYN5/WT5 SYN10/WT10*
**yMS536**	synVsynX and Y6.5	*MATa/α ura3Δ0/ura3::KanMX ho::tR(ccu)J/ho::Hyg lys2::NatMX/LYS2 SYN5/WT5 SYN10/WT10*
**yMS537**	synVsynX and Q62.5	*MATa/α ura3Δ0/ura3::KanMX ho::tR(ccu)J/ho::Hyg lys2::NatMX/LYS2 SYN5/WT5 SYN10/WT10*
**yMS538**	synVsynX and Q89.8	*MATa/α ura3Δ0/ura3::KanMX ho::tR(ccu)J/ho::Hyg lys2::NatMX/LYS2 SYN5/WT5 SYN10/WT10*
**yMS539**	synVsynX and Y9.6	*MATa/α ura3Δ0/ura3::KanMX ho::tR(ccu)J/ho::Hyg lys2::NatMX/LYS2 SYN5/WT5 SYN10/WT10*
**yMS540**	synVsynX and Q74.4	*MATa/α ura3Δ0/ura3::KanMX ho::tR(ccu)J/ho::Hyg lys2::NatMX/LYS2 SYN5/WT5 SYN10/WT10*
**yMS541**	synVsynX and Q69.8	*MATa/α ura3Δ0/ura3::KanMX ho::tR(ccu)J/ho::Hyg lys2::NatMX/LYS2 SYN5/WT5 SYN10/WT10*
**yMS542**	synVsynX and W7	*MATa/α ura3Δ0/ura3::KanMX ho::tR(ccu)J/ho::Hyg lys2::NatMX/LYS2 SYN5/WT5 SYN10/WT10*
**yMS543**	synVsynX and Q31.4	*MATa/α ura3Δ0/ura3::KanMX ho::tR(ccu)J/ho::Hyg lys2::NatMX/LYS2 SYN5/WT5 SYN10/WT10*
**yMS544**	synVsynX and Y8.5	*MATa/α ura3Δ0/ura3::KanMX ho::tR(ccu)J/ho::Hyg lys2::NatMX/LYS2 SYN5/WT5 SYN10/WT10*
**yMS545**	synVsynX and Z1.1	*MATa/α ura3Δ0/ura3::KanMX ho::tR(ccu)J/ho::Hyg lys2::NatMX/LYS2 SYN5/WT5 SYN10/WT10*
**yMS546**	synVsynX and Y8.1	*MATa/α ura3Δ0/ura3::KanMX ho::tR(ccu)J/ho::Hyg lys2::NatMX/LYS2 SYN5/WT5 SYN10/WT10*
**yMS547**	synVsynX and N-17	*MATa/α ura3Δ0/ura3::KanMX ho::tR(ccu)J/ho::Hyg lys2::NatMX/LYS2 SYN5/WT5 SYN10/WT10*
**yMS548**	**synVsynX and CBS 5829**	*MATa/α ura3Δ0/ura3::KanMX ho::tR(ccu)J/ho::Hyg lys2::NatMX/LYS2 SYN5/WT5 SYN10/WT10*
**yMS549**	synVsynX and KPN3829	*MATa/α ura3Δ0/ura3::KanMX ho::tR(ccu)J/ho::Hyg lys2::NatMX/LYS2 SYN5/WT5 SYN10/WT10*
**yMS550**	synVsynX and YPS138	*MATa/α ura3Δ0/ura3::KanMX ho::tR(ccu)J/ho::Hyg lys2::NatMX/LYS2 SYN5/WT5 SYN10/WT10*
**yMS551**	synVsynX and DBVPG 6304	*MATa/α ura3Δ0/ura3::KanMX ho::tR(ccu)J/ho::Hyg lys2::NatMX/LYS2 SYN5/WT5 SYN10/WT10*
**yMS552**	synVsynX and A12	*MATa/α ura3Δ0/ura3::KanMX ho::tR(ccu)J/ho::Hyg lys2::NatMX/LYS2 SYN5/WT5 SYN10/WT10*
**yMS553**	synVsynX and N-44	*MATa/α ura3Δ0/ura3::KanMX ho::tR(ccu)J/ho::Hyg lys2::NatMX/LYS2 SYN5/WT5 SYN10/WT10*
**yMS554**	synVsynX and IFO 1804	*MATa/α ura3Δ0/ura3::KanMX ho::tR(ccu)J/ho::Hyg lys2::NatMX/LYS2 SYN5/WT5 SYN10/WT10*

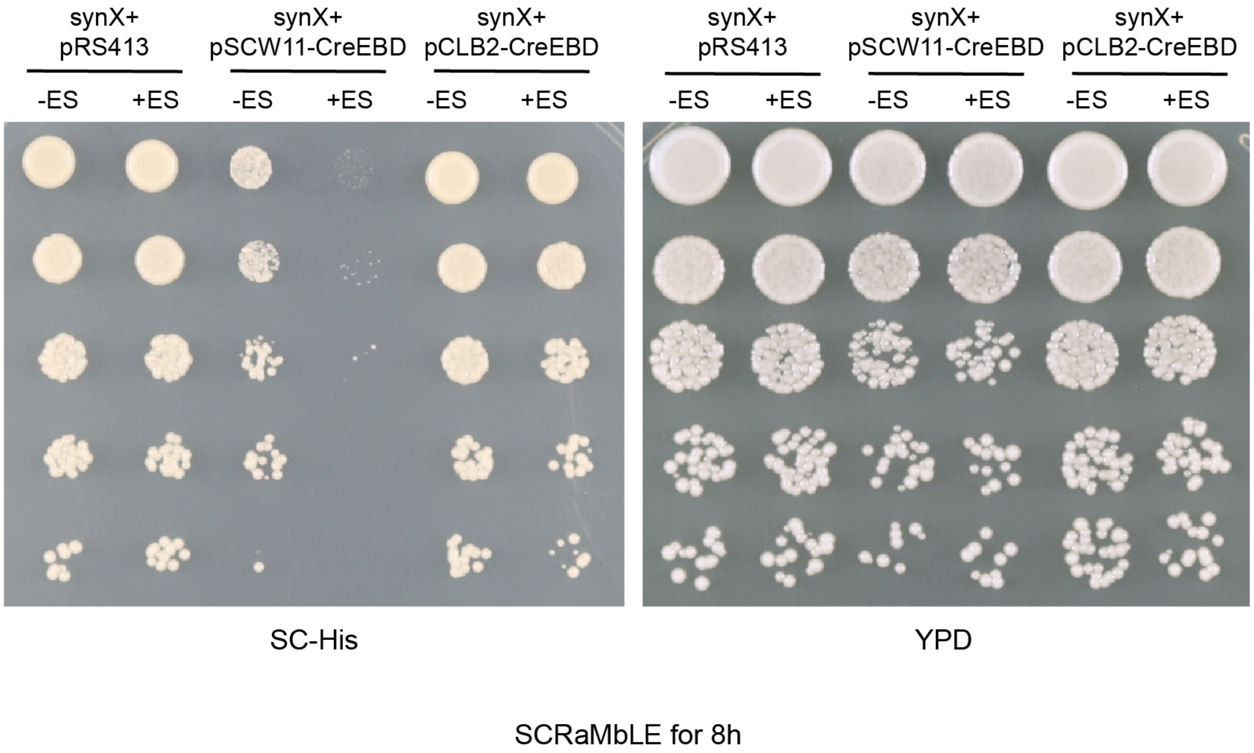

Supplementary Figure 1 Comparison of pSCW11 and pCLB2 promoters for Cre-EBD. Haploid strains bearing synX were transformed with the episomal shuttle vector pRS413 or pRS413 expressing Cre-EBD driven by either pSCW11 or pCLB2. Strains were subjected to SCRaMbLE for 8h in SC–His media without (–ES) or with 1μM β-estradiol (+ES) and were subsequently washed and plated as a serial dilution assay onto SC–His or YPD plates.

strain	location	structure variation	SCRaMbLEd segments	ORFs	size (bp)
yYW166	A	deletion	2-9	YJL222W-YJL217W	17031
	B	deletion	40-61	YJL161W-YJL130C	58298
	C	deletion	112-127	YJL052C-YJL028W	51548
	D	duplication	128-131	YJL027C-YJL022W	6544
	E	deletion	166	none	810
	F	deletion	194-248	YJR093C-YJR159W	137041
yYW167	G	deletion	47-53	YJL154C-YJL140W	20815
	H	deletion	73	none	386
	I	deletion	173-174	YJR055W-YJR056C	3548
	J	deletion	246-247	none	1047

Supplementary Figure 2 Structural variation in yYW166 (blue) and yYW167 (red) reveals a common deletion of YJL154C-YJL140W among deletions ranging from 0.38 to 137 kb in length. The duplicated segment YJL027C-YJL022W is 6.5 kb in length.

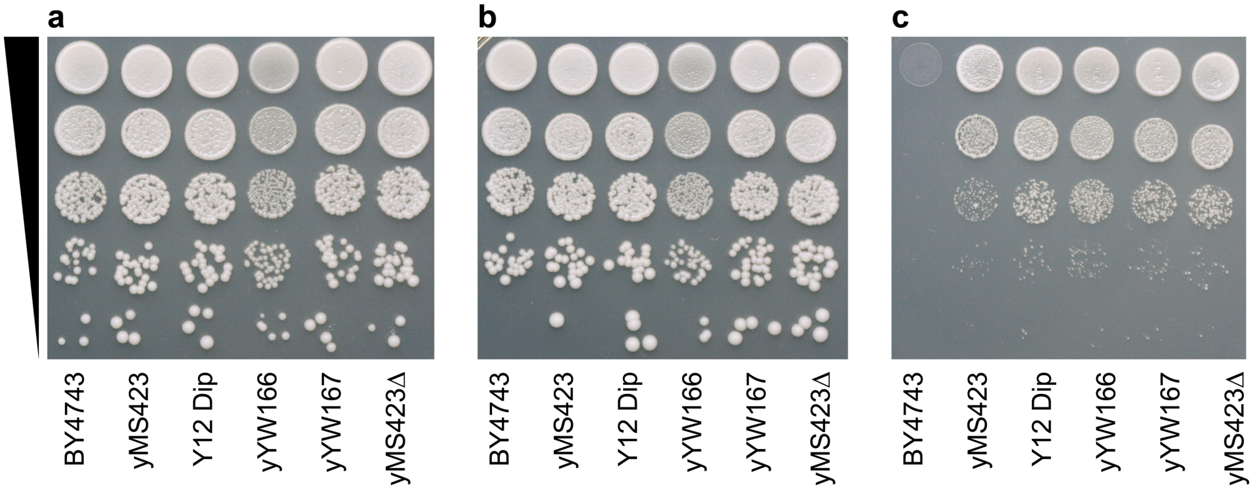

Supplementary Figure 3 Serial dilution assay showing growth on YPD of diploid strains BY4743, yMS423 (Y12-*synX* heterozygous diploid non-SCRaMbLEd parent), Y12 homozygous diploid, yYW166 (SCRaMbLEd yMS423), yYW167 (SCRaMbLEd yMS423), and yMS423Δ (yMS423 with deletion of YJL154C to YJL140W in *synX*) at (**a**) 30ºC, (**b**) 37ºC, and (**c**) 42ºC. Assays are shown after 48h of incubation.

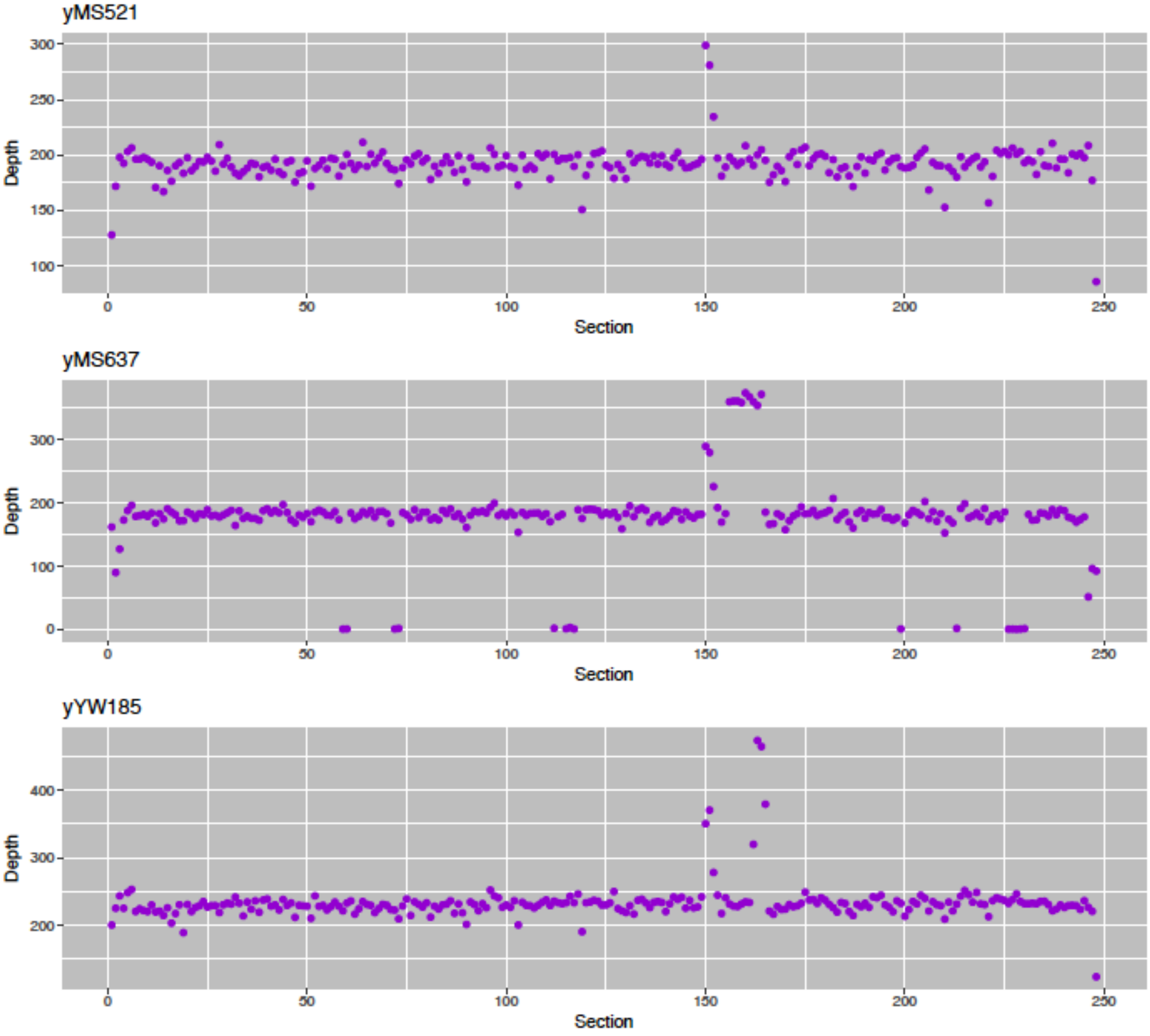

Supplementary Figure 4 Whole genome sequencing analysis revealed a common duplication region in both the SCRaMbLEd strains yMS637 and yYW185 compared to their non-SCRaMbLEd parent yMS521. This region, spanning two loxPsym-flanked segments and just over 2 kb in length, contains only the *POL32* gene.

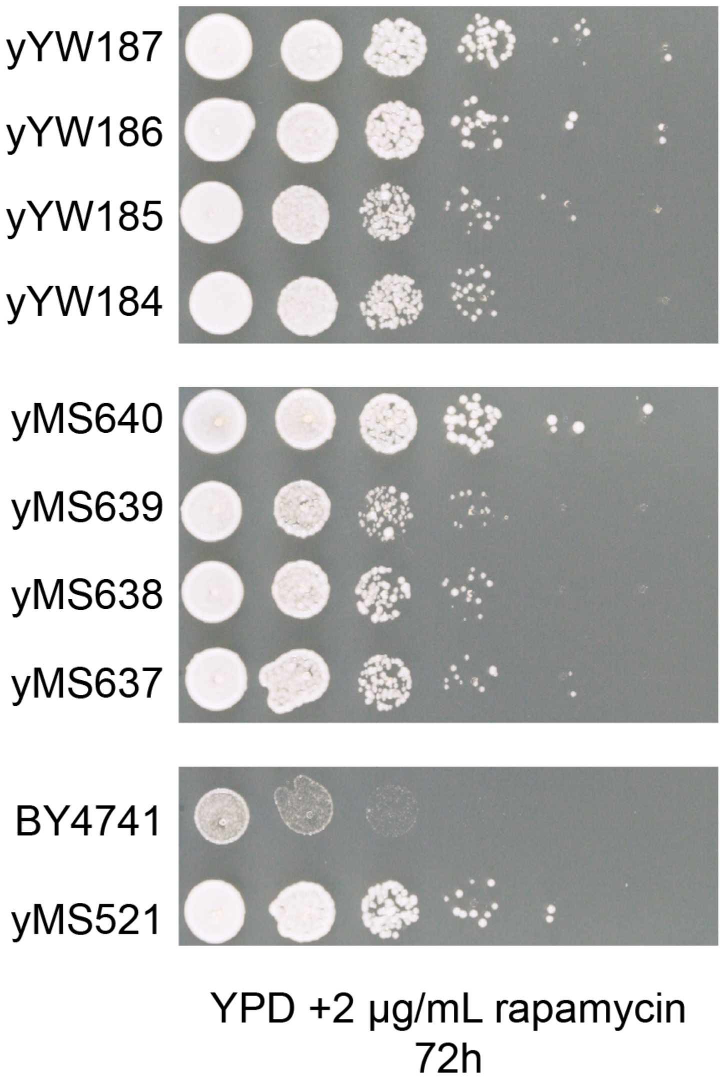

Supplementary Figure 5 Growth of SCRaMbLEd, caffeine-tolerant CBS5829-*synX* and CBS5829-*synVsynX* strains in rapamycin.

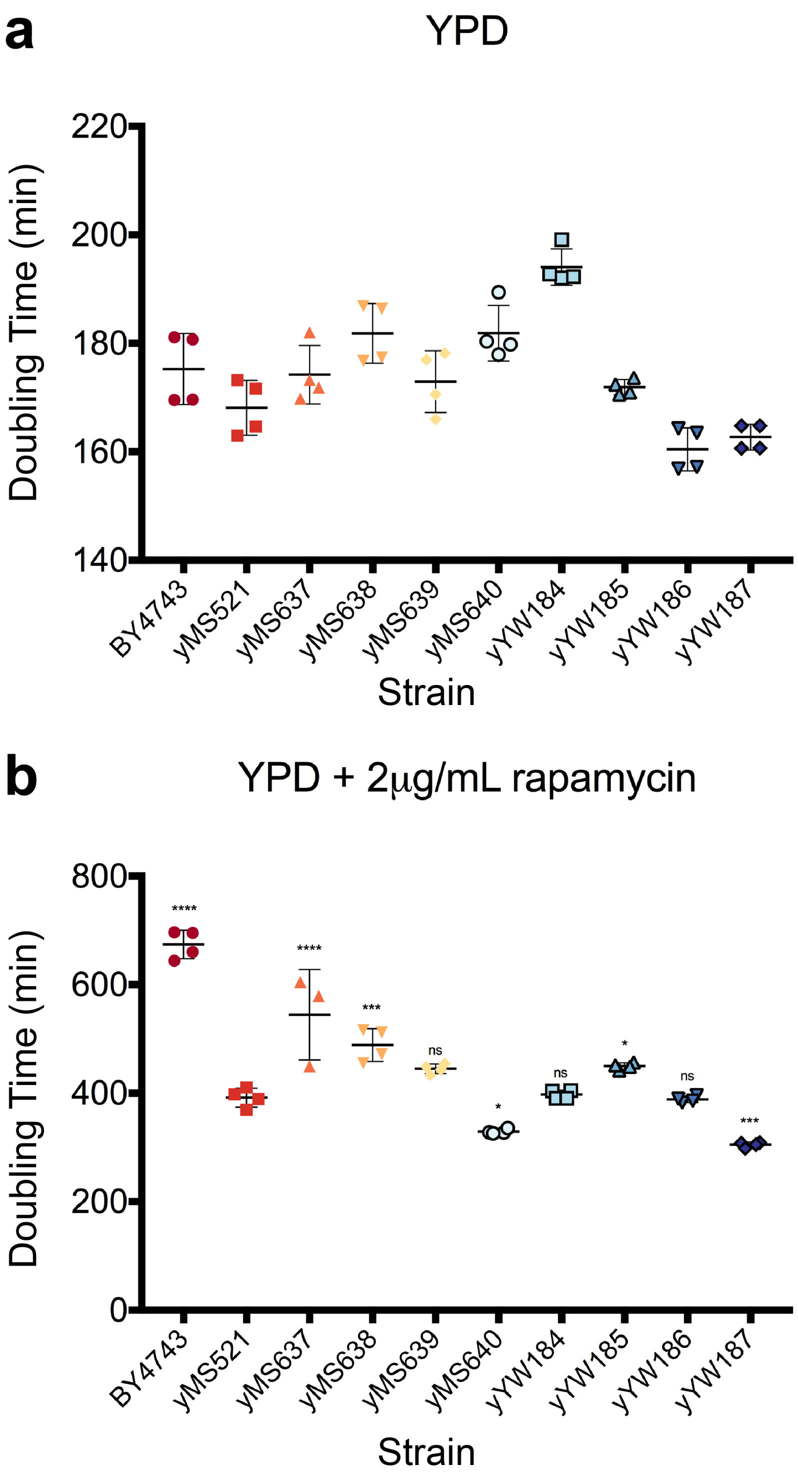

Supplementary Figure 6 BY4743, yMS521, yMS637, yMS638, yMS639, yMS640, yYW184, yYW185, yYW186, and yYW187 were all grown in liquid YPD overnight and diluted to a starting A_{600} of 0.1 in either YPD or YPD with 2 μg/mL rapamycin. These strains were then cultured in a 96-well plate reader with shaking. Optical density measurements were taken every 10m and used to calculate doubling time. Error bars shown are mean and standard deviation from four technical replicates (three in the case of yMS637 in YPD with 2 μg/mL rapamycin). One-way ANOVA with multiple comparisons was used to assess difference between each sample and yMS521 (****: $p < 0.0001$, ***: $p < 0.001$, *: $p < 0.05$, ns: not significant).

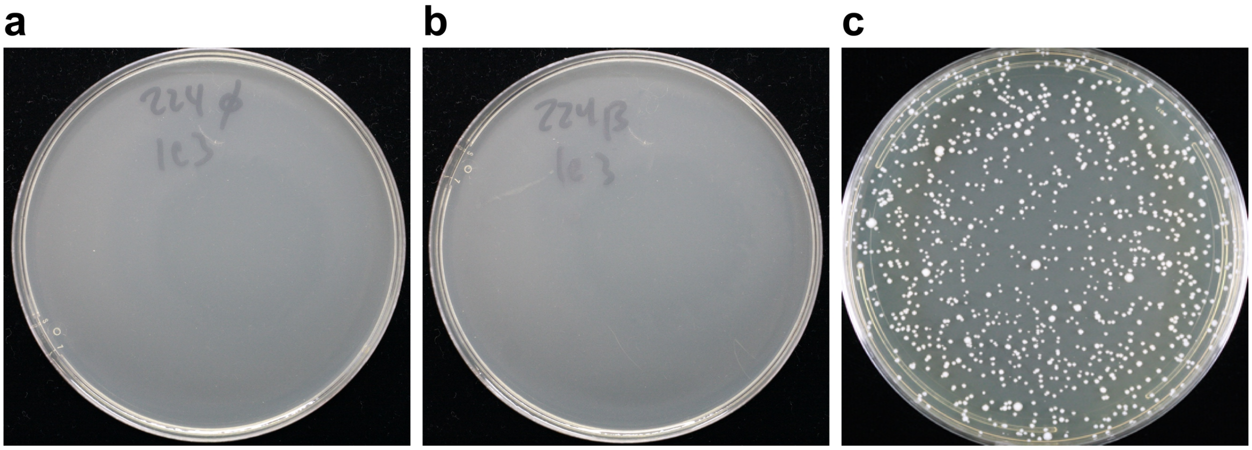

Supplementary Figure 7 Growth after 96h of (**a**) non-SCRaMbLEd S288C-*synX* cells at 42ºC, (**b**) SCRaMbLEd S288C-*synX* cells at 42ºC, (**c**) SCRaMbLEd Y12-*synX* cells at 42ºC. Large colonies from the plate in (**c**) were picked for further analyses.

Supplementary Table 2: Variants shared between strains yMS637 and yYW185

Chromosome	Coordinate	Original	Variant	Notes
**BY4741_chr01**	27072	A	G	FLO9
**BY4741_chr01**	27075	G	A	FLO9
**BY4741_chr01**	27090	T	C	FLO9
**BY4741_chr01**	27099	T	C	FLO9
**BY4741_chr01**	27105	A	G	FLO9
**BY4741_chr01**	27126	T	C	FLO9
**BY4741_chr01**	27129	C	G	FLO9
**BY4741_chr08**	475929	TTA	T	low coverage
**BY4741_chr13**	65927	C	CTGT	NUP188
**BY4741_chr14**	522403	T	C	YNL054W-B transposable element
**BY4741_chr14**	522427	C	T	YNL054W-B transposable element
**BY4741_chr14**	522481	T	C	YNL054W-B transposable element
**BY4741_chr14**	522550	A	G	YNL054W-B transposable element
**BY4741_chr14**	522560	A	G	YNL054W-B transposable element
**CBS5829.chr10**	341311	TA	T	similar to Ty in S288C
**CBS5829.chr10**	707973	C	CAT	not in ORF
**CBS5829.chr01**	4837	CT	C	not in ORF
**CBS5829.chr01**	11602	A	AG	not in ORF
**CBS5829.chr01**	11948	G	A	YAL059C dubious ORF
**CBS5829.chr01**	16801	AGACATGGACT	A	GPB2
**CBS5829.chr01**	18213	A	T	not in ORF
**CBS5829.chr01**	21569	T	C	synonymous
**CBS5829.chr01**	24921	G	A	synonymous
**CBS5829.chr01**	26814	T	C	synonymous
**CBS5829.chr01**	30444	C	T	synonymous
**CBS5829.chr01**	34182	A	G	PTA1 K-->E
**CBS5829.chr11**	67624	T	C	MNN4 K-->R
**CBS5829.chr11**	67666	C	T	MNN4 R-->K
**CBS5829.chr12**	264204	ATT	A	low coverage
**CBS5829.chr12**	453171	G	GAT	low coverage
**CBS5829.chr13**	560747	A	T	not in ORF
**CBS5829.chr13**	780322	C	CA	not in ORF
**CBS5829.chr13**	798914	GA	G	not in ORF
**CBS5829.chr14**	634155	T	C	synonymous
**syn10**	232000	C	CA	not in ORF

The CBS5829 genome was downloaded from the Saccharomyces Genome Resequencing Project (http://www.sanger.ac.uk/research/projects/genomeinformatics/sgrp.html), while the *synX* sequence used was yeast_chr10_9_01 from BioStudio (http://54.160.105.26/gbrowse2/)

Supplementary Table 3: Inferred rearrangements in SCRaMbLEd strains

strain	segment 1	segment 2	depth	inferred rearrangement
**yM637**	10.129	10.-96	52	inversion
	10.131	10.97	52	
	10.-113	10.-111	52	deletion of segment 112
	10.-231	10.-225	51	deletion of segments 226-230
	10.-61	10.-58	47	deletion of segments 59 and 60
	10.-132	10.13	45	inversion
	10.-74	10.-71	45	deletion of segments 72 and 73
	10.212	10.214	43	deletion of segment 213
	10.-118	10.-114	43	deletion of segments 115-117
	10.164	10.156	35	duplication of segments 156-164
	10.-200	10.-198	22	deletion of segment 199
	10.02	10.-245	21	inversion
	10.-03	10.-01	13	deletion of segment 2
	10.245	10.247	13	deletion of segment 246
**yMS638**	10.-17	10.1	54	inversion
	10.16	10.-09	52	inversion
	10.-121	10.-119	44	deletion of segment 120
**yMS639**	10.-231	10.224	78	inversion
	10.-17	10.-15	52	deletion
	10.23	10.-223	40	inversion
**yYW186**	5.51	5.42	41	in parental strain
	5.116	5.-112	37	inversion
	5.-117	5.113	32	inversion
**yYW187**	10.-97	10.82	45	inversion
	5.51	5.42	36	in parental strain
	10.96	10.-81	26	inversion
**yYW195**	5.-48	5.-46	69	deletion
	5.12	5.07	56	deletion
	5.51	5.42	52	in parental strain
	5.-45	5.-43	46	deletion
**yYW196**	5.51	5.42	59	in parental strain
